# The Effect of Static Trial on Knee Adduction Moment During Walking

**DOI:** 10.7759/cureus.69157

**Published:** 2024-09-11

**Authors:** Omar Althomali

**Affiliations:** 1 Department of Physiotherapy, University of Hail, College of Applied Medical Sciences, Hail, SAU

**Keywords:** 3d gait analysis, adduction moment, kinematics, kinetics, static trial

## Abstract

Background and aim

Sophisticated technologies in rehabilitation, such as three-dimensional gait analysis, allow for measuring kinematic and kinetic variables while performing activities. The first peak external knee adduction moment (EKAM) is considered an important outcome in individuals with knee osteoarthritis (OA) and has been shown to be affected by changes in foot position in static trials. The present study aimed to explore the variables in static trials that may lead to changes in the value of the EKAM while walking.

Methods

Twelve individuals participated in the current study and were asked to perform three static trials as follows: 20° toe-out, straight (0°), and 20° toe-in. The participants were asked to walk five trials (their own shoes and paces). The first peak EKAM was the main study outcome and was compared between conditions. Linear regression was used to investigate which variables in the static trials significantly predicted the magnitude of change in the EKAM while walking.

Results

The first peak EKAM significantly decreased by 8.2% while walking when changing the foot position in static trials from 20° toe-in to 20° toe-out. The magnitude of change in the EKAM was significantly (p<0.01) predicted by the magnitude of change in the knee joint frontal plane angle, shank transverse plane angle, ankle joint frontal plane angle, and hip joint frontal plane angle during static trials between 20° toe-in and 20° toe-out. The model was able to predict 94% of the variation in the EKAM due to changes in foot position during static trials.

Conclusion

Modifications in foot position during static trials led to a change in the first peak EKAM while walking. Researchers should focus on controlling the knee joint frontal plane angle, shank transverse plane angle, ankle joint frontal plane angle, and hip joint frontal plane angle during static trials when conducting longitudinal or crossover studies. Controlling these variables is necessary to reduce the likelihood of the EKAM being affected by static trials and to ensure that the EKAM changes in dynamic trials are not masked or increased by static trials.

## Introduction

The knee is recognized as the joint most affected by osteoarthritis (OA) and is considered a global disease [1]. The societal and economic burden of knee OA is substantial and is expected to increase over time [2-4]. Based on the International Classification of Functioning, Disability, and Health (ICF), OA causes limitations and restrictions in performing physical activities [5]. In Saudi Arabia, recent studies have shown an increased prevalence of knee OA in the population [6,7]. Interestingly, OA was categorized as a serious disease in 2016 by the Osteoarthritis Research Society International [8].

Previous studies have found that the medial knee compartment is affected more severely than the lateral compartment [9-12]. This has been attributed to several reasons, such as load distribution. The medial compartment bears a higher load during activities such as stair climbing and walking compared to the lateral compartment [13]. In the first 20% of the gait cycle, the medial compartment transmits 70% of the weight [14]. This higher load is caused by the line of gravity as it passes the medial to the knee joint center. The gold standard method for measuring joint loading is through a knee implant tray, which is invasive and impractical [15].

A non-invasive method was developed to estimate joint loading using a gait analysis system. Three-dimensional gait analysis is a sophisticated technology that can measure kinetic and kinematic outcomes during activities [16]. The external knee adduction moment (EKAM) has been shown to have a strong correlation with joint loading [17]. Furthermore, the EKAM is considered a valid surrogate for joint loading [17-19]. Supporting evidence demonstrated that the EKAM was linked with higher knee OA pain, with the odds ratio of pain increasing by 3.05 with every 1% body weight and height (BW·Ht) increment in the EKAM [20]. The risk of progression in knee OA was found to increase approximately six times with a one-unit increase in the EKAM [21], and a significant correlation was observed between the EKAM and the loss of knee cartilage over one year [22]. This highlights the importance of the EKAM in research and understanding the disease.

Several studies have revealed higher EKAMs in individuals affected with OA compared to the control groups [18,23]. In contrast, research has not consistently found any significant difference in the EKAM between individuals affected by knee OA and healthy participants [24,25]. This contradiction was highlighted by a systematic review and meta-analysis, which revealed inconsistencies between studies using the EKAM in individuals with knee OA [26]. Several factors have been suggested as causes of these differences, including the degree of disease progression [25,27], walking approach [28], and inconsistency in marker placement or other related methodological causes affecting the EKAM [29].

A 2023 study conducted by our research group investigated the effect of changing foot position during static trials on the EKAM of the same dynamic walking trials [30]. The results showed that changing the foot position from 20° toe-out to 20° toe-in significantly changed the EKAM by 8.2% while using the same dynamic trials. The findings pointed to a change in the knee adduction angle as a possible cause of the observed variation. However, the exact cause of this change in the EKAM remains unknown. Since the EKAM is an important outcome in research, it is imperative to understand what variables affect it when changing foot position. Therefore, the present study aimed to investigate the static trial variables that affect the knee adduction moment during walking.

## Materials and methods

The current study is a crossover randomized trial that obtained ethical approval from the Research Ethics Committee at the University of Hail (#H-2020-229). Eligible participants had to be able to walk independently without assistance, have no deformity in the lower limbs, and have not suffered any lower limb injuries in the previous three years. Individuals with any neurological or neuromuscular impairment affecting their walking ability were not allowed to participate in the study. Potential participants were given time to consider their involvement and signed a consent form after receiving a full explanation of the study. In the present work, the Vicon system (Oxford, UK: Oxford Metrics) was used for the three-dimensional gait analysis and consisted of two force platforms (Watertown, NY: Advanced Mechanical Technology Incorporation {AMTI} force plate, type OR67) synchronized with eight cameras (Vicon-Bonita infrared motion cameras; Oxford Metrics: Oxford, UK). The sampling rate for the cameras was 100 Hz, and that for the force platform was 1000 Hz.

The sample size for the current study was considered to be adequate since this study analyzed data retrospectively from our previous publication [30]. The sample size was calculated using a method similar to a previous study [31]. The G power application was used to calculate the sample size for the first peak EKAM with 95 power, effect size 0.52, and alpha error 0.05. The observed power for repeated measure ANOVA for the main study outcome (first peak EKAM) was 0.78 which shows a good power.

The lab was prepared and calibrated based on the manufacturer’s manual before the participants arrived. After changing into a T-shirt and shorts, the markers and casts were attached to the skin based on the calibrated anatomical system technique (CAST) [32]. Previous research indicates that the CAST model is superior to other models and effectively reduces skin artifacts [32]. Markers (1.45 cm) were attached to the following bony landmarks: anterior and posterior superior iliac spines, iliac crest, greater trochanters, lateral and medial condyles, lateral and medial malleoli, first, second, and fifth metatarsal heads, and the most prominent part of the heel. Four casts (rigid cluster plates) were attached to the shanks and thighs in the midline on the anterolateral view. Each cluster contained four markers attached to each corner of the rectangle.

In the CAST model, to analyze the dynamic trial, a static pose needs to be collected, which is used to define the joint center. During the static trials, the participants were asked to stand in three poses: 20° toe-in, 0° (neutral), and 20° toe-out. These poses were used to evaluate the same dynamic walking trials. The conditions (static poses) were randomized in a crossover pattern using a randomization block (www.randomization.com). The selected pose angles were based on previous studies showing that individuals affected by OA may walk with their toes out up to 26°, while healthy individuals were able to walk within 13°-25° toe-in and toe-out from their normal standing baseline [33,34].

**Figure 1:**
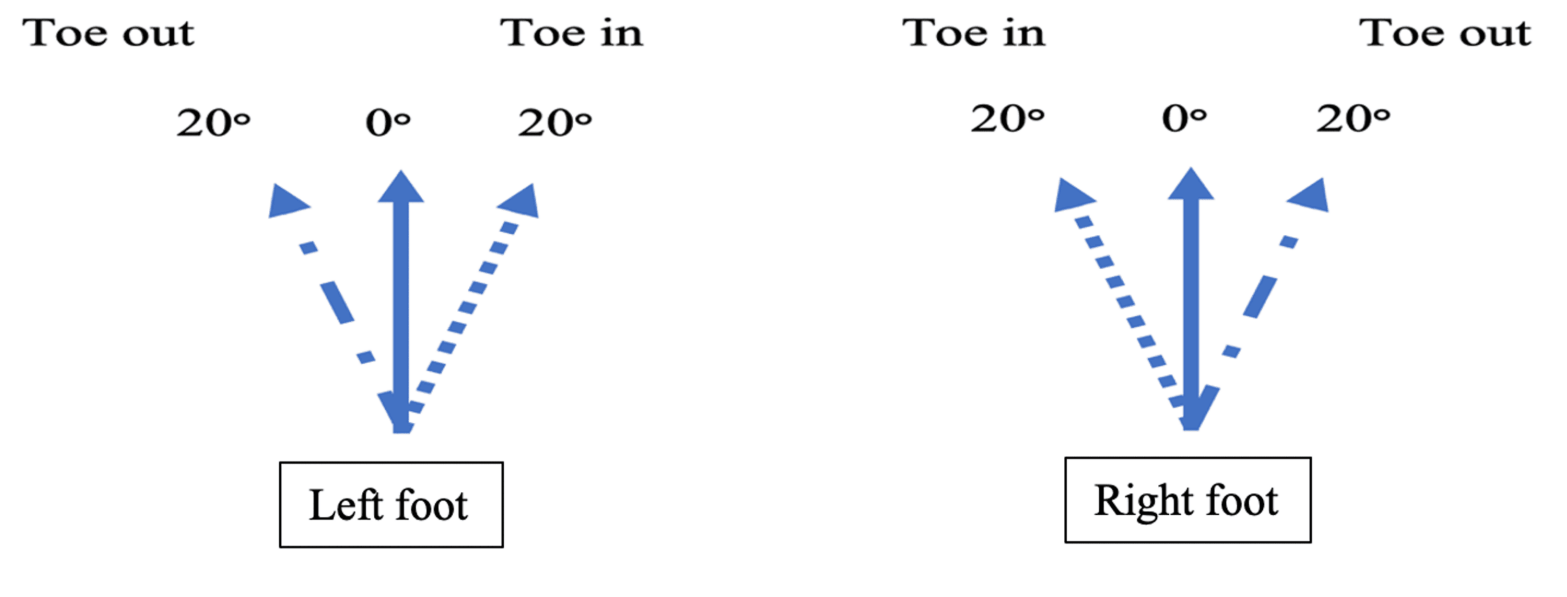
Feet placement during the static trials. Dotted line = 20° toe-in, solid line = 0°, and dash-dot line = 20° toe-out.

To ensure consistency and accuracy in placing the participant in the required foot position, a custom protractor was created on the force platform using tape (Figure 1). Previous research has used a similar method to guide participants while collecting data [25-27]. Each participant was instructed to position the second toe and the heel at the angle marked on the floor and hold the position for 10 seconds. A 30-second rest was given between each static trial and the next.

After performing the static trials, each participant was asked to complete five successful walking trials at their everyday walking speed. For a trial to be considered successful, the participant had to walk naturally at their usual speed and each foot had to be in full contact with the force platform, with no part of the foot on the edge. The participants were not made aware of the force platform to prevent them from adjusting their walking pattern to target it.

The collected data were processed using Vicon Nexus software (Oxford, UK: Vicon Motion Systems) as the first step. The markers were labeled and exported as V3D files to be processed in Visual 3D (Germantown, MD). In this software, a six-degree model was built, defining the hip joint center based on a regression model from a previous study [35]. The knee and ankle joint centers were defined as the midpoints between the medial and lateral markers of the condyle and malleoli, respectively. The invasive dynamic theory was used to calculate the moment throughout segment geometric and inertial properties were estimated based on the previous method [30,31]. A proximal coordinate system was used to resolve the moment. The trials were interpolated and subsequently filtered at 25 Hz for kinetics and 6 Hz for kinematics. The moment was expressed as an external moment after being normalized to body mass. The main outcome of the current study is the EKAM at the first peak, based on our previous findings showing significant changes between the 20° toe-in and 20° toe-out conditions [30]. The first peak EKAM was calculated as the maximum value between 0% and 33% of the stance phase.

Data analysis

The static trials were analyzed to investigate which variable(s) led to significant changes in the first peak EKAM between 20° toe-in and 20° toe-out. The following variables were exported from these trials (20° toe-in, 0° {neutral}, 20° toe-out): hip, knee, and ankle joints sagittal plane angles; hip, knee, and ankle joints frontal plane angles; hip, knee, and ankle joints transverse plane angles; thigh, shank, foot, and pelvic sagittal plane angles; thigh, shank, foot, and pelvic frontal plane angles; and thigh, shank, foot, and pelvic transverse plane angles. All previously mentioned outcomes in the static trials were compared using a repeated-measure ANOVA between conditions. The results revealed significant changes in the hip sagittal plane angle, hip frontal plane angle, hip transverse plane angle, knee frontal plane angle, knee transverse plane angle, ankle frontal plane angle, ankle transverse plane angle, thigh frontal plane angle, thigh transverse plane angle, shank transverse plane angle, and foot transverse plane angle (appendix). Since there was a significant difference in the first peak EKAM only between 20° toe-in and 20° toe-out, the analysis focused solely on these two conditions. The magnitude of change in the first peak EKAM was calculated between 20° toe-in and 20° toe-out. The magnitude of change was also calculated for the statistically significant outcomes of the static trials by subtracting the value of each variable at 20° toe-out from 20° toe-in.

Statistical analysis

A stepwise linear regression model was used to investigate which changes in static variables led to the effect on the first peak EKAM between 20° toe-in and 20° toe-out. All variables (magnitude of change) that were significant between static trials were entered into the model as dependent variables, while the magnitude of change in the first peak EKAM was the independent variable. The significance level was considered to be p<0.05.

## Results

**Figure 2:**
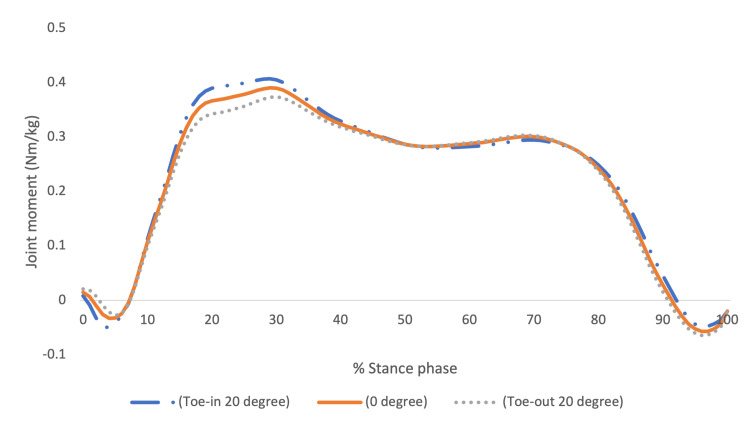
The knee adduction moment during walking. Dotted line = 20° toe-in, solid line = 0°, dash-dot line = 20° toe-out.

The 12 participants in the current study had an average walking speed of 0.99 (±0.1) m/s. The demographic characteristics of the participants were as follows: height of 1.73 (±0.05) meters, weight of 70.93 (±15.46) kg, age of 23.5 (±2.91) years, and body mass index of 23.55 (±4.41) kg/m^2^. The mean values of the first peak EKAM during walking in 20° toe-in, 0°, and 20° toe-out are presented in Table 1 and Figure 2. A significant difference was observed in the first peak EKAM between conditions, and a pairwise comparison showed a significant change between 20° toe-in and 20° toe-out. The mean difference in the first peak EKAM between 20° toe-in and 20° toe-out was 0.04 Nm/kg.

**Table 1:** Linear regression results for the association between the magnitude of change in EKAM and the magnitude of change in variables in static trials. EKAM: external knee adduction moment

Variable	20° toe-in	0°	20° toe-out	ANOVA	Effect size (partial η^2^)
				p-Value	
First peak EKAM (Nm/kg)	0.44 (±0.11)	0.43 (±0.1)	0.41 (±0.1)	0.01	0.41
Pairwise comparison adjusted to Bonferroni correction					
Conditions	Conditions	Mean difference	p-Value	Lower bound	Upper bound
20° toe-in	0°	0.02	0.06	0.001	0.04
	20° toe-out	0.04	0.04	0.001	0.07
0°	20° toe-out	0.02	0.11	0.004	0.04

The mean magnitude of change between 20° toe-in and 20° toe-out in the hip joint sagittal plane angle, hip joint frontal plane angle, hip joint transverse plane angle, knee joint frontal plane angle, knee joint transverse plane angle, ankle joint frontal plane angle, ankle joint transverse plane angle, thigh frontal plane angle, thigh transverse plane angle, shank transverse plane angle, and foot transverse plane angle are presented in Table 2. A linear regression analysis revealed that the magnitude of change in knee joint frontal plane angle, shank transverse plane angle, ankle joint frontal plane angle, and hip joint frontal plane angle during static trials between 20° toe-in and 20° toe-out significantly predicted the magnitude of change in first peak EKAM, F(4, 7) = 41.391, p<0.01 (Table 3). The previously mentioned variables accounted for 94% (adjusted R square) of the explained variability in the amount of change in the first peak EKAM. Model number four was the final and chosen model since it explained more of the variation in the magnitude of change in first peak EKAM between 20° toe-in and 20° toe-out.

**Table 2:** Magnitude of changes in static trial variables between 20° toe-in and 20° toe-out.

Variable	Mean	SD
Hip joint sagittal plane angle	2.81°	3.23°
Hip joint frontal plane angle	1.55°	2.34°
Hip joint transverse angle	21.15°	8.43°
Knee joint frontal plane angle	1.52°	0.88°
Knee joint transverse angle	2.76°	1.56°
Ankle joint frontal plane angle	1.60°	1.88°
Ankle joint transverse plane angle	8.85°	5.77°
Thigh frontal plane angle	1.55°	2.09°
Thigh transverse plane angle	21.11°	8.39°
Shank transverse plane angle	23.96°	7.75°
Foot transverse plane angle	32.76°	5.79°

**Table 3:** Linear regression results for the association between the magnitude of change in EKAM and the magnitude of change in variables in static trials. EKAM: external knee adduction moment

Model	Unstandardized coefficients		Standardized coefficients	t-test	Significance	95.0% confidence interval for B	
	B	Beta				Lower bound	Upper bound
Constant	0.050	0.015	-	3.436	0.011	0.016	0.085
Knee joint frontal plane angle	0.042	0.005	0.848	9.256	<0.01	0.032	0.053
Shank transverse plane	0.003	0.001	0.521	5.463	<0.01	0.002	0.004
Ankle joint frontal plane angle	0.021	0.003	0.907	7.579	<0.01	0.015	0.028
Hip joint frontal plane angle	0.010	0.002	0.504	4.149	<0.01	0.004	0.015

## Discussion

Our previous study showed that changing foot position during static trials led to a significant change in the EKAM during walking [30]. The current study aimed to investigate which changes in static trials led to changes in the EKAM during walking. The results show that during static trials, the frontal plane hip, knee and ankle joint angles, and shank transverse plane angle affect the EKAM. This finding has clinical implications in studies that use the three-dimensional gait analysis system to investigate within-subject changes. Therefore, the previously mentioned variables should be controlled during a static trial to avoid any unreal changes in the EKAM.

The magnitude of change in the first peak EKAM during walking between 20° toe-in and 20° toe-out was 0.04 (0.04) Nm/kg. This is equivalent to an 8.2% reduction in the EKAM when moving the foot in a static position from 20° toe-in to 20° toe-out. This change has a large effect size, which highlights the importance of controlling foot position during static trials. Several studies have found that individuals with medial knee OA have higher EKAMs than the control groups [13,18,36]. In contrast, a previous study showed no significant differences in the EKAM between healthy individuals and individuals with medial knee OA [37]. It was found that the knee adduction moment was significantly correlated with medial knee loading and the EKAM [14,17]. The odds ratio in pain was also shown to increase by 3.05 when the difference in the EKAM between limbs was equal to or higher than 1.0% BW·Ht [20]. Moreover, a previous study estimated a 6.46 times increase in the risk of OA progression with one unit of increase in the EKAM [21,22]. Therefore, the evidence discussed shows the importance of the EKAM as a biomechanical variable in individuals with knee OA.

In the management of knee OA, non-surgical treatment is considered first-line, such as biomechanical treatment like the use of lateral wedge insoles, which are cheap and easy to use [38]. A previous study showed a reduction in EKAM by 5.85% with the use of lateral wedge insoles in individuals with knee OA [39]. This reduction is comparable to the current study, which showed a reduction in EKAM by 8.2% when changing the foot position during a static trial from internal rotation to external rotation. This highlights the importance of such an effect, which may lead to accentuating or reducing the real effect of any treatment on EKAM.

A previous study highlighted several possible sources of error in calculating the moment during clinical trials, including modeling assumptions and measurement errors [40]. Therefore, understanding the source of error is considered an important priority if we are to reduce inconsistency between studies and reach accurate clinical interpretations. The cause of the reduction in the EKAM with changing the foot position from 20° toe-in to 20° toe-out was predicted by the current study. Ninety-four percent of the variation in the EKAM is attributed to differences in the following variables during static trials: knee joint frontal plane angle, shank transverse plane angle, ankle joint frontal plane angle, and hip joint frontal plane angle. Therefore, during static trials, frontal plane alignment in the hip, knee, and ankle joints is an important factor to be considered to avoid misleading results and inappropriate clinical interpretations.

Similar effects on the moment were observed when using different marker locations to define the coordination system used in the gait analysis [41]. Specifically, different marker configurations were utilized to define the joint center (medial and lateral epicondyles, medial and lateral condyles, and tibial ridges). The results showed significant changes in the knee joint moments (flexion and extension). Furthermore, a previous study investigated the effect of hip position during static trials on joint kinematics [42]. The result revealed a significant effect on kinematics by changing the hip position during the static trial. Although the study investigated the effect on joint angle, changes in moment value are expected, since the calculations of moment depend on the joint angle and other factors, which is consistent with the current study.

Limitations and further directions

While the present work offers valuable insights into the effect of changing foot position during static trials on the EKAM, several limitations should be noted. First, we included only healthy males, which may limit the generalizability of the findings and highlight an opportunity for future studies. Second, although the inverse kinetic theory was employed to analyze the joint in the proximal coordinate system, it is not clear whether the distal coordinate system may suffer similar effects on the EKAM with changes in foot position during static trials. Although we have investigated the impact of foot position on EKAM while walking, further research is needed to determine if other activities, such as stair climbing and descending, might show similar effects.

## Conclusions

The current study’s findings clearly demonstrate that several factors in static trials may lead to unreal changes in the EKAM while walking. The reduction in the EKAM, found to be equal to 0.04 (0.04) Nm/kg (8.2%) when moving the foot during static trials from 20° toe-in to 20° toe-out, was predicted by the regression model. This model identified four variables that accounted for up to 94% of the change in the EKAM with changes in foot position during static trials. The magnitude of change in the knee joint frontal plane angle, shank transverse plane angle, ankle joint frontal plane angle, and hip joint frontal plane angle was significantly linked to the magnitude of change in the EKAM. This highlights the importance of using standardization methods while conducting within-subject designs, especially considering the aforementioned four variables in static trials. Future studies should consider standardizing foot position during static trials to reduce measurement errors and achieve accurate clinical interpretations in longitudinal studies.

## Appendices

**Table 4:** Mean (SD) for joint kinetics variables during static trials.

Variable	Toe-in 20°	0°	Toe-out 20°	p-Value	Effect size
Hip sagittal plane angle	11.26 (8.16)	8.89 (8.28)	8.44 (7.81)	<0.01	0.362
Hip frontal plane angle	-0.09 (2.50)	-1.41 (2.77)	-1.65 (2.61)	0.031	0.271
Hip transverse plane angle	6.04 (9.57)	-6.73 (9.83)	-15.11 (5.42)	<0.01	0.843
Knee sagittal plane angle	2.26 (6.06)	1.67 (6.46)	0.73 (6.01)	0.174	0.147
Knee frontal plane angle	-1.04 (3.84)	-.20 (3.72)	0.48 (3.86)	<0.01	0.696
Knee transverse plane angle	-2.27 (2.96)	-3.33 (2.79)	-5.03 (2.54)	<0.01	0.719
Ankle sagittal plane angle	5.27 (2.88)	5.24 (3.51)	4.20 (2.49)	0.173	0.147
Ankle frontal plane angle	-0.22 (2.51)	1.17 (2.69)	1.38 (2.24)	<0.01	0.351
Ankle transverse plane angle	2.59 (9.78)	-0.75 (8.64)	-6.25 (5.38)	<0.01	0.668
Thigh sagittal plane angle	-3.16 (4.17)	-3.79 (4.14)	-3.87 (4.75)	0.427	0.074
Thigh frontal plane angle	0.85 (2.88)	-0.44 (2.74)	-0.70 (2.41)	0.011	0.334
Thigh transverse plane angle	6.05 (9.57)	-6.67 (9.79)	-15.06 (5.37)	<0.01	0.844
Shank sagittal plane angle	-5.37 (2.93)	-5.46 (3.90)	-4.39 (2.68)	0.218	0.129
Shank frontal plane angle	0.05 (2.21)	0.07 (1.77)	0.24 (2.06)	0.877	0.012
Shank transverse plane angle	3.94 (7.27)	-9.87 (7.88)	-20.02 (5.35)	<0.01	0.886
Foot sagittal plane angle	7.2888E-05 (0.00087414)	0.000156 (0.00088053)	0.00069347 (0.0017737)	0.123	0.198
Foot frontal plane angle	1.5041E-05 (0.00302235)	-0.0010583 (0.00445768)	-0.0011473 (0.00552484)	0.746	0.026
Foot transverse plane angle	6.56 (5.49)	-10.53 (4.39)	-26.20 (4.66)	<0.01	0.951
Pelvic sagittal plane angle	-14.42 (6.05)	-12.69 (5.67)	-12.32 (5.20)	0.051	0.268
Pelvic frontal plane angle	-0.91 (1.22)	-0.95 (1.37)	-0.94 (1.44)	0.987	0.001
Pelvic transverse plane angle	-0.22 (0.36)	-0.15 (0.28)	-0.17 (0.29)	0.328	0.096

**Table 5:** Pairwise comparison between kinetics variables during the different static trials.

Variable	Conditions	Conditions	Mean difference	Standard error	p-Value	95% Confidence interval for difference	
						Lower bound	Upper bound
Hip sagittal plane angle	Toe-in 20°	0°	2.363	0.654	0.012	0.517	4.208
		Toe-out 20°	2.814	0.932	0.035	0.185	5.443
	0°	Toe-out 20°	0.451	0.950	1.000	-2.228	3.130
Hip frontal plane angle	Toe-in 20°	0°	1.316	0.357	0.011	0.309	2.322
		Toe-out 20°	1.553	0.677	0.127	-0.355	3.462
	0°	Toe-out 20°	0.238	0.663	1.000	-1.631	2.107
Hip transverse plane angle	Toe-in 20°	0°	12.773	0.470	<0.01	11.447	14.099
		Toe-out 20°	21.151	2.433	<0.01	14.291	28.011
	0°	Toe-out 20°	8.378	2.321	0.012	1.834	14.922
Knee frontal plane angle	Toe-in 20°	0°	-.835	0.208	<0.01	-1.422	-0.247
		Toe-out 20°	-1.521	0.255	<0.01	-2.241	-0.801
	0°	Toe-out 20°	-0.686	0.173	<0.01	-1.175	-0.197
Knee transverse plane angle	Toe-in 20°	0°	1.058	0.328	0.024	0.133	1.984
		Toe-out 20°	2.761	0.451	<0.01	1.489	4.033
	0°	Toe-out 20°	1.703	0.320	<0.01	0.800	2.606
Ankle frontal plane angle	Toe-in 20°	0°	-1.382	0.337	<0.01	-2.333	-0.432
		Toe-out 20°	-1.595	0.543	0.041	-3.127	-0.064
	0°	Toe-out 20°	-0.213	0.590	1.000	-1.877	1.451
Ankle transverse plane angle	Toe-in 20°	0°	3.340	0.857	<0.01	0.922	5.757
		Toe-out 20°	8.848	1.666	<0.01	4.150	13.547
	0°	Toe-out 20°	5.509	1.377	<0.01	1.624	9.393
Thigh frontal plane angle	Toe-in 20°	0°	1.298	0.404	0.025	0.160	2.437
		Toe-out 20°	1.551	0.604	0.078	-0.152	3.255
	0°	Toe-out 20°	0.253	0.475	1.000	-1.086	1.591
Thigh transverse plane angle	Toe-in 20°	0°	12.725	0.478	<0.01	11.376	14.074
		Toe-out 20°	21.111	2.422	<0.01	14.282	27.941
	0°	Toe-out 20°	8.386	2.297	0.011	1.908	14.864
Shank transverse plane angle	Toe-in 20°	0°	13.813	0.656	<0.01	11.964	15.662
		Toe-out 20°	23.958	2.237	<0.01	17.649	30.266
	0°	Toe-out 20°	10.144	2.179	<0.01	3.999	16.289
Foot transverse plane angle	Toe-in 20°	0°	17.091	1.317	<0.01	13.376	20.807
		Toe-out 20°	32.763	1.671	<0.01	28.051	37.474
	0°	Toe-out 20°	15.671	1.756	<0.01	10.720	20.623
